# THE STRONGERMEMORY PROGRAM: EXPLORING COGNITIVE BENEFITS AND FOSTERING COMMUNITY

**DOI:** 10.1093/geroni/igad104.2882

**Published:** 2023-12-21

**Authors:** Catherine Tompkins, Emily Ihara, Francesca Keesee, Mckenzie Lauber, Catherine Magee, Jessica Fredericksen, Robert Liebreich

**Affiliations:** George Mason University, Fairfax, Virginia, United States; George Mason University, Fairfax, Virginia, United States; George Mason University, Fairfax, Virginia, United States; George Mason University, Fairfax, Virginia, United States; George Mason University, Fairfax, Virginia, United States; Goodwin Living, Alexandria, Virginia, United States; Goodwin Living, Alexandria, Virginia, United States

## Abstract

**Background:**

StrongerMemory is a brain health program centered on spending 30 minutes per day on handwritten journaling, reading aloud, and arithmetic exercises as a method to enhance cognition in older adults. The StrongerMemory research protocol is a 12-week program that includes a weekly meeting. After 104 participants completed the StrongerMemory research protocol, they were invited to participate in a focus group to share their experiences. Six focus groups were conducted by members of the StrongerMemory research team yielding 30 participants.

**Methods:**

Focus groups were conducted virtually, with a set of pre-determined, open-ended questions centering around participant experiences and attitudes towards the StrongerMemory program. Participants were also asked to reflect on differences in their memory, cognition and well-being before and after StrongerMemory participation. Two researchers independently coded the focus group transcripts, and utilized the grounded theory analysis techniques of memoing and constant comparative analysis to explore the data. Common themes were then discussed.

**Results:**

Five overarching themes emerged: Motivating, appreciating, challenging, committing, and enhancing. Conceptualizations of these themes focused on participants’ experiences and suggestions for strengthening the program. “Fostering community” was an outcome of the program often discussed.

**Conclusion:**

The participant experience in StrongerMemory revealed unique perspectives on their motivation for participation and provided the researchers with new insights into the program such as fostering community within older adult groups. Further research includes exploring cognitive and social benefits of group participation in the StrongerMemory program.

